# *TaFlo2-A1*, an ortholog of rice *Flo2*, is associated with thousand grain weight in bread wheat (*Triticum aestivum* L.)

**DOI:** 10.1186/s12870-017-1114-3

**Published:** 2017-10-16

**Authors:** Muhammad Sajjad, Xiaoling Ma, Sultan Habibullah Khan, Muhammad Shoaib, Yanhong Song, Wenlong Yang, Aimin Zhang, Dongcheng Liu

**Affiliations:** 1 State Key Laboratory of Plant Cell and Chromosome Engineering, Institute of Genetics and Developmental Biology, Chinese Academy of Sciences, 1 West Beichen Road, Chaoyang District, Beijing, 100101 China; 2 Department of Environmental Sciences, COMSATS Institute of Information Technology, Vehari, 61100 Pakistan; 3 University of Chinese Academy of Sciences, Beijing, 100049 China; 4 U.S.-Pakistan Center for Advanced Studies in Agriculture and Food Security (US-PCAS-AFS), University of Agriculture Faisalabad, Faisalabad, 38040 Pakistan; 5 College of Agronomy, The Collaborative Innovation Center of Grain Crops in Henan, Henan Agricultural University, 63 Nongye Road, Zhengzhou, 450002 China

**Keywords:** *Floury endosperm*, Haplotype variation, Gene cloning, TGW, *Triticum aestivum*

## Abstract

**Background:**

The *Flo2* gene is a member of a conserved gene family in plants. This gene has been found to be related to thousand grain weight (TGW) in rice. Its orthologs in hexaploid wheat were cloned, and the haplotype variation in *TaFlo2-A1* was tested for association with TGW.

**Results:**

The cloned sequences of *TaFlo2-A1*, *TaFlo2-B1* and *TaFlo2-D1* contained 23, 23 and 24 exons, respectively. The deduced proteins of *TaFlo2-A1* (1734 aa), *TaFlo2-B1* (1698 aa) and *TaFlo2-D1* (1682 aa) were highly similar (>94%) and exhibited >77% similarity with the rice FLO2 protein. Like the rice FLO2 protein, four tetratricopeptide repeat (TPR) motifs were observed in the deduced *TaFLO2* protein. An 8-bp InDel (−10 to −17 bp) in the promoter region and five SNPs in first intron of *TaFlo2-A1* together formed two haplotypes, *TaFlo2-A1a* and *TaFlo2-A1b*, in bread wheat. *TaFlo2* was located on homeologous group 2 chromosomes. *TaFlo2-A1* was inferred to be located on deletion bin ‘2AL1–0.85-1.00’. The *TaFlo2-A1* haplotypes were characterized in the Chinese Micro Core Collection (MCC) and Pakistani wheat collection using the molecular marker TaFlo2-Indel8. *TaFlo2-A1* was found to be associated with TGW but not with grain number per spike (GpS) in both the MCC and Pakistani wheat collections. The frequency of *TaFlo2-A1b* (positive haplotype) was low in commercial wheat cultivars; thus this haplotype can be selected to improve grain weight without negatively affecting GpS. The expression level of *TaFlo2-A1* in developing grains at 5 DAF (days after flowering) was positively correlated with TGW in cultivars carrying the positive haplotype.

**Conclusion:**

This study will likely lead to additional investigations to understand the regulatory mechanism of the *Flo2* gene in hexaploid wheat. Furthermore, the newly developed molecular marker ‘TaFlo2-InDel8’ could be incorporated into the kit of wheat breeders for use in marker-assisted selection.

**Electronic supplementary material:**

The online version of this article (10.1186/s12870-017-1114-3) contains supplementary material, which is available to authorized users.

## Background

Enhancing the grain yield potential of wheat is a key focus of wheat breeders. Grain yield is the product of various yield components. Wheat grain yield per unit area is the product of grain yield per spike (GYS) and the number of spikes per unit area. The latter depends on sowing density and is highly affected by environmental factors. The GYS is determined by grain number per spike (GpS) and thousand grain weight (TGW), which are variably correlated in different wheat collections/populations. A significant negative correlation between these two traits has been reported in bi-parental populations [1–3], but no significant correlation was observed between TGW and GpS in collections of Chinese landraces [4], French winter wheat cultivars [5] and CIMMYT-derived spring wheat collections [6]. On the other hand, a significant positive correlation between TGW and GpS was reported in modern Chinese cultivars [4].

TGW in wheat has been one of the target traits for selection during domestication and breeding [7, 8]. For example, in China, an increase in wheat yield potential from ~1 T ha^−1^ in 1992 to ~5.4 T ha^−1^ today is mainly due to the genetic increase in TGW from ~20 g to ~45 g, respectively [9]. However, genetic gain in TGW has not reached its limit and thus provides an opportunity to increase yield potential [9]. It is estimated that an increase in yield of 140–160 kg ha^−1^ can be obtained by just a 1-g increase in TGW [10]. However, genes and their roles in controlling TGW in wheat are still largely unknown. In wheat, TGW is a quantitative trait controlled by several genes/QTL distributed on all chromosomes [8, 11]. For example, Su et al. [12] discovered eight TGW-related QTL on chromosomes 2D, 4B, 5A, 7A and 7B, explaining up to 16.2% of the phenotypic variation. Similarly, four QTL for TGW on chromosomes 1D, 2A, 5D, and 6A explained 5.9 to 20.1% of phenotypic variation in different environments [13].

Nevertheless, from this plethora of QTLs, few loci/genes have been cloned by map-based cloning approaches mainly because of the large and complex hexaploid genome (~17 Gb) that consists of three homeologous genomes (A, B, D) and an abundance of repeat sequences (80%) [14]. Studies on comparative genomics have shown high synteny and collinearity among different grass genomes, such as those of wheat, barley, rice, millet, maize and sorghum. This pattern of genome organization in the members of the grass family provides a powerful approach for gene discovery in common wheat [15]. A large number of genes have been discovered in common wheat by synteny-based cloning, in which the gene sequences of model crops such as rice and barley have been used as references to identify orthologous genes in wheat. For example, the genes *TaTGW6* [16, 17], *TaCwi-A1* [18], *TaSus2-2B* [19], *TaSus2-2A*, *TaSus1-7A* [20], *TaGW2-6A*, *6B* [9, 12, 21], *TaCKX6-D1* [22], *TaSAP1-A1* [23], *TaGS1a* [24], *TaGS-D1* [25], and *TaGASR-A1* [26] were discovered using rice-wheat synteny and using molecular markers in marker-assisted wheat breeding. Hence, the isolation and characterization of genes controlling grain size in common wheat will help breeders maximize yield potential by establishing gene-based breeding programs.

The *FLOURY ENDOSPERM2* (*Flo2*) gene is a member of a conserved gene family in plants. In rice, this gene has been shown to have a tetratricopeptide repeat (TPR) motif consisting of 3–16 tandem repeats of 34 aa residues that mediate protein–protein interactions in the nucleus [27, 28]. The *OsFlo2* gene was cloned in the *indica* cultivar ‘Kasalath’; this gene was found to have 23 exons and 22 introns and coded for a protein consisting of 1720 amino acid residues that had three TPR motifs in the middle [27]. The expression of *Flo2* was constitutive in both vegetative tissues and developing seeds, and the expression was relatively high level in developing seeds. The *flo2* mutants exhibit a significant reduction in amylose content and grain weight and exhibit altered expression of various starch synthesis-related genes, indicating its key role in regulating rice grain weight and starch quality [27, 28]. In this article, we report the rice-wheat synteny-based isolation of *Flo2* orthologs in hexaploid wheat, the association of *TaFlo2-A1* sequence polymorphisms with TGW and the comparison of temporal expression profiles of *TaFlo2-A1* haplotypes in flag leaves and developing caryopses.

## Methods

### Plant materials

For cloning the *TaFlo2* gene in hexaploid wheat, Chinese Spring (CS) and two sets of cultivars with lower and higher TGW were used; the set of cultivars with higher TGW included Dixiuzao (49.5 g), Enmai4 (49.2 g), Liying 5(49.4 g) and Laizhou 953 (52.2 g), and the set with lower TGW included Jinyang 60 (23.5 g), Baihuamai (24.1 g), Sanyuehuang (25.2 g) and Zipi (25.5 g). The Chinese Spring nulli-tetrasomic lines were used to assign *TaFlo2* genes to wheat homeologous chromosomes. The Chinese Micro Core Collection (MCC, 262 accessions) and Pakistani wheat collection (130 accessions) were used to confirm the association between *TaFlo2-A1* haplotypes and TGW. To avoid the effect of population structure, normalized MCC subpopulations were used for association analysis [19, 29]. The Pakistani collection was selected based on previous reports [30, 31] considering the effect of population structure on association analyses.

### Cloning and characterization of *TaFlo2* sequences

The genomic sequence of the rice *OsFlo2* gene (NC_008397) was used as a query for BLAST searches against the wheat sequences database in the URGI (https://urgi.versailles.inra.fr/). All wheat scaffold sequences with high similarity to the rice *OsFlo2* sequence were assembled to construct a putative *TaFlo2* gene using DNAMAN (http://www.lynnon.com). Based on the scaffold sequences, six conserved primer pairs were used to specifically amplify *TaFlo2* coding and promoter sequences from the three wheat sub-genomes: A, B and D (Table 1). The *TaFlo2* mRNA of 4902 bp was cloned in Chinese Spring using three primer pairs designed from the predicted mRNA sequence (Table 1). Genomic DNA was extracted from young seedlings of each line using the CTAB method [32]. A 20-μl reaction volume comprising 0.5 μl (5 μM) of each primer, 2× Taq mix (GenStar, Beijing, China) and 100 ng of DNA was used for PCR amplification that consisted of a cycle profile of 5 min at 94 °C; 35 cycles of 30 s at 94 °C, 30 s at 60 °C and 4 min at 72 °C; and a final extension of 10 min at 72 °C. The PCR products were detected by electrophoresis in 1% agarose gels with nucleic acid dye (Tiangen, Beijing, China), and gel images were captured using a UV spectrometer (BioRad, Hercules, CA, USA). The targeted PCR products were obtained from the agarose gels and purified using the TIANgel MIDI Purification Kit (Tiangen, Beijing, China). The purified PCR products were then ligated into the pGEM-T Easy cloning vector (TransGen Biotech, Beijing, China). The ligation product was transformed to 50 μl of Trans1-T1 competent cells by the heat shock method (Tiangen, Beijing, China). Positive clones from each transformation were selected based on positive PCR tests and were sequenced (Beijing Genomics Institute). The sequences were analyzed using DNAMAN software (http://www.lynnon.com).

**Table 1:** Primer sequences used in this study

Primer name	Primer sequence (5′-3′)	Position on scaffold sequence	Annealing temperature (°C)	PCR product size	Function
Flo2-1F	TGTGCTGGAATCACCCACTC	793–812	60	1061	cloning TaFlo2 /polymorphism detection
Flo2-1R	GCGCGGCGAAAACTAATCAT	1853–1844			
Flo2-2F	GTGCCGTCCATAATCGTTGC	1546–1565	60	1781	cloning TaFlo2 /polymorphism detection
Flo2-2R	CATGTGCGGCAAAAGACACA	3326–3307			
Flo2-3F	AACGGGCATGTGTCTTTTGC	3299–3318	60	3025	cloning TaFlo2 /polymorphism detection
Flo2-3R	CGACGCAGCTCTGAAAATCG	6332–6313			
Flo2-4F	CGCTTAGCAGTGGATTTGCC	5719–5738	60	3948	cloning TaFlo2
Flo2-4R	ATCCAACAAACAGGTGCCCA	9667–9647			
Flo2-5F	TTGCGGAAGCCCATCATTCT	8387–8406	60	3836	cloning TaFlo2
Flo2-5R	TGACCTTCTGCGGATGCTTT	1222–12,203			
Flo2-6F	CAGAACAGGGCCGGTACAAT	11,368–11,387	60	2600	cloning TaFlo2
Flo2-6R	CGCTCATCTGGATAGGGCAA	13,967–13,948			
TaFlo2-InDel8F	ACCCCTCCTCCGTTATCGTC	1337–1356	60	145/153	8-bp InDel polymorphism in *TaFlo2-A1*
TaFlo2-InDel8R	CCTCCTTCTTCTTGCGGTCG	1470–1489			
Flo2-A1F	GTGCTCCGATCCGATGTGCAGTTAT	5387–5411	58	587	2A specific
Flo2-A1R	GTGCACAACCAAGTAAAAGG	5973–5954			
Flo2-B1F	GTC ATC ACTAGAGGA ATTTTCC	6851–6872	58	902	2B specific
Flo2-B1R	CTCTCAGAACTGTGGAT	7752–7736			
Flo2-D1F	CTGTATCTGTAATTTGTTCCG	5378–5398	58	326	2D specific
Flo2-D1R	CTTCCGAAAAATGTGGGG	5704–5687			
mFlo2-1F	TAACGGTGGTGCACTTGTGT	–	58	1868	Cloning mRNA
mFlo2-1R	TCAGCCGCAAGTTATGCTCA	–			
mFlo2-2F	TGCGGACGAGATGGAAAACA	–	58	1809	Cloning mRNA
mFlo2-2R	AGCAGTCAGCCGATGGTATG	–			
mFlo2-3F	ATGCGTACTCCCTAAGCGTG	–	58	1889	Cloning mRNA
mFlo2-3R	CACGAAGTGCTGCTTGCTTT	–			
eTaFlo2F	CCATTCGGCTTTCGTGCAAA	–	55	134	Expression analysis
eTaFlo2R	TGTTTTCCATCTCGTCCGCA	–			
ActinF	AGCCATACTGTGCCAATC	–	55	134	Internal control
ActinR	GCAGTGGTGGTGAAGGAGTAA	–			

### Characterization of *TaFlo2-A1* haplotypes and development of haplotype-specific markers

The 262 MCC and 130 Pakistani varieties were genotyped with the primer pair TaFlo2-InDel8, and PCR product was run on 8% polyacrylamide gels. Based on TaFlo2-InDel8 scoring, the MCC and Pakistani accessions sorted into two groups according to their haplotypes (*TaFlo2-A1a* or *TaFlo2-A1b*) for the *TaFlo2-A1* gene. For MCC, the average values of TGW of the two haplotype groups were calculated using replicated data collected from 3 years (2002, 2005, 2006) of plants in Beijing [19]. For Pakistani varieties, the average values of TGW of the two haplotype groups were calculated using replicated data from 2 years (2009, 2010) of field trials at the University of Agriculture, Faisalabad. The resulting values were then compared and statistically analyzed using SPSS 13.0 for Windows (IBM, New York, USA).

### Quantitative RT-PCR analysis of *TaFlo2-A1* haplotypes

The Yangmai 19, Chinese Spring, Pubing3228, Shannong23 and Zhengmai9405 varieties were sown at the experimental station of the Institute of Genetics & Developmental Biology, CAS in Beijing, China in October 2014; three rows of each variety were planted. The length of each row was 2 m, and the row-to-row distance was 20 cm. The plants were managed in accordance with standard agronomic practices; irrigation and fertilizer were supplied for optimal growth. Twelve- day-old flag leaves of five plants from each variety were harvested and stored at −80 °C. Unfertilized grains were collected from each variety 1–2 days before flowering (DBF). Fertilized grains were collected from each variety at 5, 10, 15, 20 and 25 days after flowering (DAF). The flag leaf and developing grain samples were processed for the preparation of total RNA as described previously [33]. Three biological replicates that were collected from different plants were analyzed separately for each variety for quantitative RT-PCR evaluation. For *TaFlo2-A1* transcripts analysis, the primer set eTaFlo2, which is specific for *TaFlo2-A1* (Table 1), was designed and used. Quantitative RT-PCR was then carried out as described by Feng et al. [34]. The wheat actin gene was used as an internal control. The relative expression level of *TaFlo2-A1* in each flag leaf and in each sample of developing grains was calculated using the data of three technical replicates as described previously [35]. Statistical comparisons of *TaFlo2-A1* expression levels (presented as the mean ± SD) among different samples were made by ANOVA using SPSS 13.0.

### Bioinformatics comparison of nucleotide and protein sequences

Nucleotide and protein identities among the compared sequences were calculated using DNAMAN (http://www.lynnon.com). Amino acid sequence alignment was accomplished using ClustalW2 in EMBLEBI (www.ebi.ac.uk/Tools/msa/clustalw2). Potential signal peptide sequences in the deduced proteins of TaFLO2 and its homologs were predicted using Softberry software (http://www.softberry.com/berry.phtml). The predicted TaFLO2 protein was BLASTed both in the NCBI smart blast system (http://blast.st-va.ncbi.nlm.nih.gov/smartblast) to search for homologous proteins and in the NCBI CD system (https://www.ncbi.nlm.nih.gov/Structure/cdd/wrpsb.cgi) to search for conserved domains.

## Results

### Cloning and characterization of *TaFlo2* genes

To select potential candidate *TaFlo2* genes, the rice *Flo2* sequence (NC_008397) was used as a query against the wheat genome sequences database in the URGI (https://www.ncbi.nlm.nih.gov/Structure/cdd/wrpsb.cgi). Three bread wheat scaffolds (IWGSC_chr2AL_ab_k71_contigs_longerthan_200_6436403, IWGSC_chr2BL_ab_k71_contigs_longerthan_200_7959819, IWGSC_chr2DL_ab_k71_contigs_longerthan_200_9909583) with high similarity (*E* value = 0 and similarity >73%) were identified as potential orthologs to the rice *Flo2* gene. The sequences of these scaffolds were downloaded and assembled with DNASTAR (http://www.dnastar.com/) to construct a putative *TaFlo2* sequence.

**Fig. 1:**
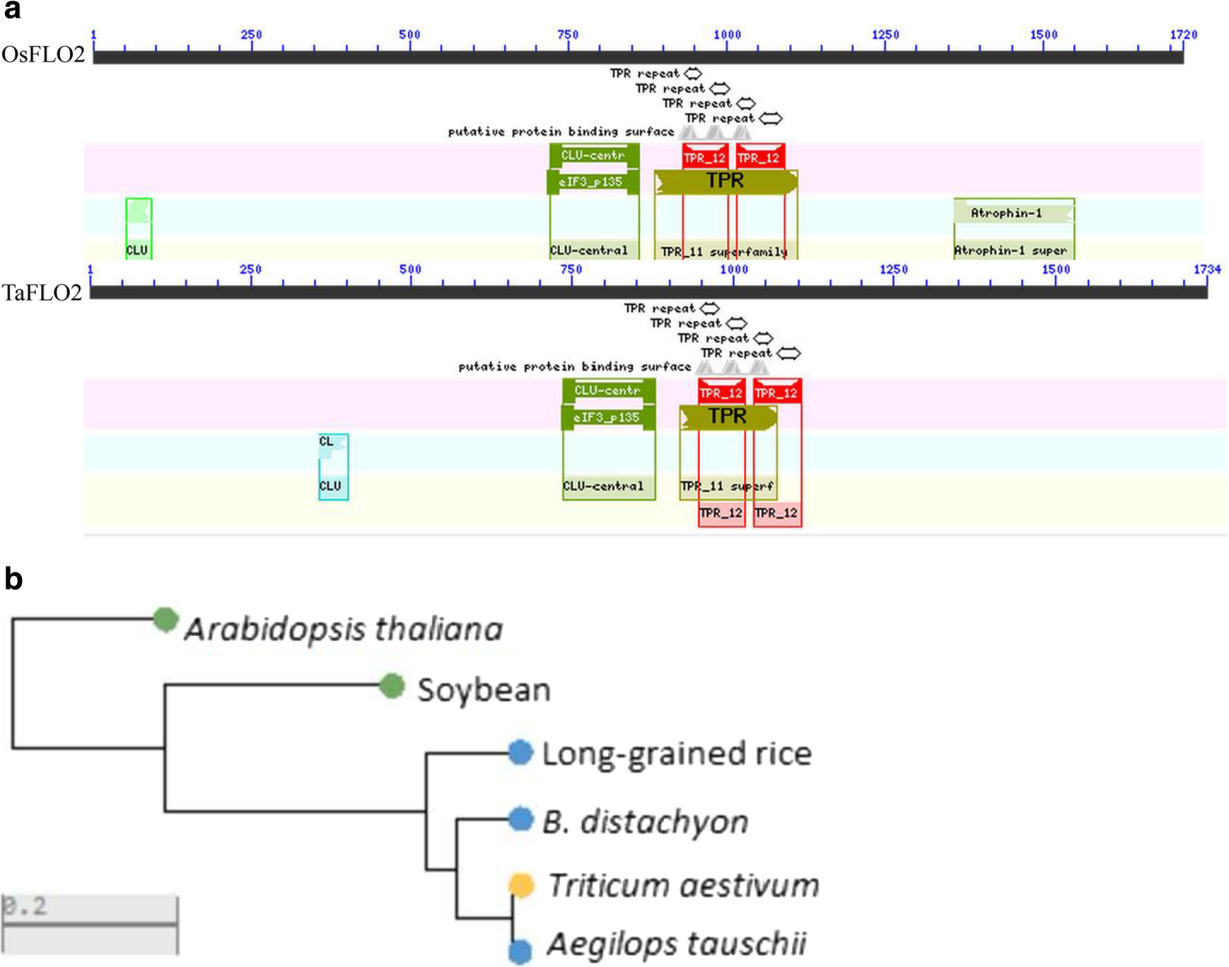
**a** Putative structure of the OsFLO2 and TaFLO2 proteins. Clu_N (mitochondrial function, CLU-N-term), CL (clustered mitochondria domain), CLU-center (an uncharacterized central domain of CLU mitochondrial proteins), TPR (tetratricopeptide repeat). **b** Similarity between the TaFLO2 protein and proteins from related plant species

To search *TaFlo2* homologs and predict their deduced protein sequence and structure, Softberry (http://www.softberry.com/berry.phtml) and NCBI (https://www.ncbi.nlm.nih.gov/) tools were used. The deduced proteins of *TaFlo2-A1* (1734 aa), *TaFlo2-B1* (1698 aa) and *TaFlo2-D1* (1682 aa) were highly similar (>94% identity among themselves) and exhibited >77% similarity with the rice FLO2 protein. Like in the rice FLO2 protein [27], four tetratricopeptide repeat (TPR) motifs were observed in the deduced TaFLO2 protein at the positions of 947–988, 1032–1072, 944–1017 and 1028–1106 amino acid residues. Furthermore, three mitochondrial CLU domains were also observed at 737–878, 50–162 and 357–401 amino acid residues (Fig. 1a). The TaFLO2 protein showed high similarity with *Aegilops tauschii*, *Brachypodium distachyon* and long-grain rice proteins (Fig. 1b).

**Fig. 2:**
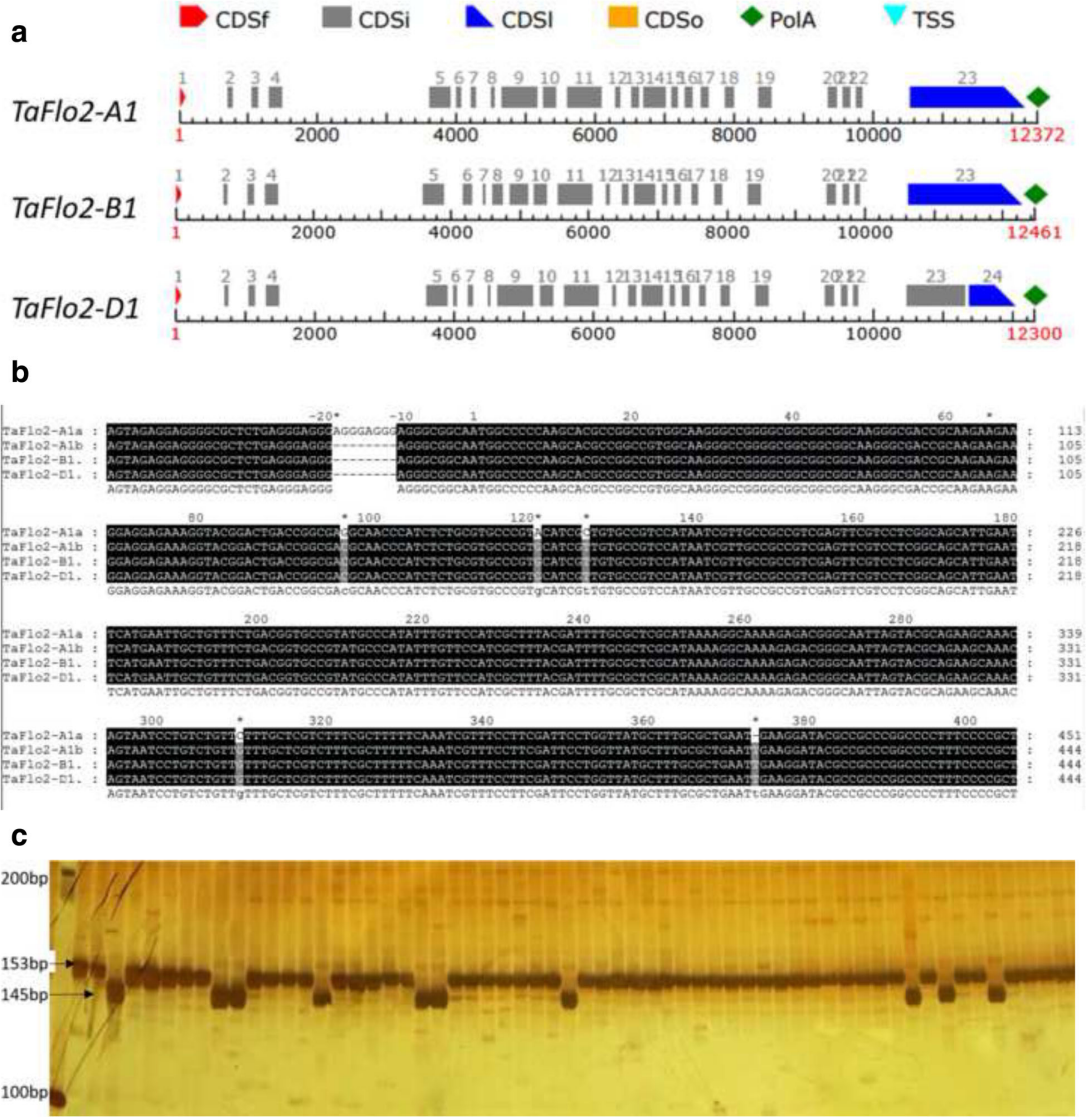
Polymorphism, molecular marker and ORF structure of *TaFlo2* homoeologs. **a** Exon and intron pattern in the ORFs of *TaFlo2-A1*, *TaFlo2-B1* and *TaFlo2-D1*. The length of each ORF (between the start and stop codons) is shown to the right of the graph. The number of nucleotides in each exon or intron is indicated. CDSf: First coding sequence, CDSi: Internal coding sequence, CDSl: Last coding sequence. **b** Alignment of the part of cloned *TaFlo2* orthologs. Polymorphism both in the promoter and first intron is indicated by stars. **c** PCR product of molecular marker TaFlo2-InDel8 discriminated by PAGE in 60 MCC accessions

To clone the full-length genomic sequence of *TaFlo2* in Chinese Spring, six conserved primer pairs were used (Table 1). The assembly of sequences with the six conserved primer pairs yielded three fragments, 14,009, 14,078 and 13,814 bp. Based on alignment with wheat scaffolds in the database and genome-specific primers, the fragments were designated *TaFlo2-A1*, *TaFlo2-B1* and *TaFlo2-D1*. The open reading frames of *TaFlo2-A1*, *TaFlo2-B1* and *TaFlo2-D1* were 12,183 bp, 12,270 bp and 12,022 bp in length, respectively. The *TaFlo2* mRNA of 4902 bp was cloned with three primer pairs (Table 1). Based on the prediction and alignment with cloned mRNA, the cloned genomic sequences from 2AL, 2BL and 2DL consisted of 23, 23 and 24 exons, respectively (Fig. 2a). Among the three homoeologs, the sequence and size of the first four exons were conserved, whereas the size and sequence of the other exons varied.

### Polymorphism detection in *TaFlo2-A1*

To detect polymorphisms in the putative *TaFlo2* sequences between high and low TGW accessions, three conserved primers that covered scaffold segments from 793 to 6332 bp were used (Table 1). Polymorphism in *TaFlo2-A1* sequences between high and low TGW accessions was observed between 1396 to 1791 bp, while no sequence variation was observed between high and low TGW accessions in *TaFlo2-B1* and *TaFlo2-D1* (Fig. 2b; Additional file 1: Figure S1). The conserved sequences of *TaFlo2-B1* and *TaFlo2-D1* in all the higher and lower TGW accessions implicated non-functional nature of these genes. An 8-bp InDel was identified in *TaFlo2-A1* sequences from 1396 to 1403 bp which was −17 to −10 bp upstream of the first coding sequence (ATG) at position 1417–1419 bp (Fig. 2b). Five SNPs (G/C, A/G, C/T, C/G and −/T) were observed at 1514, 1538, 1545, 1727 and 1791 bp. From the start codon (ATG), the positions of these five SNPs (G/C, A/G, C/T, C/G and −/T) were in the first intron at 98, 122, 128, 311 and 375 bp, respectively (Fig. 2b). The 8-bp InDel and the five SNPs together formed the two haplotypes designated *TaFlo2-A1a* and *TaFlo2-A1b* (Fig. 2b). From the position 1792 to 6332 bp, no polymorphism was observed in the *TaFlo2-A1* sequence.

### Molecular marker development and characterization of *TaFlo2-A1* haplotypes

To characterize the observed *TaFlo2-A1* haplotypes in large wheat populations, a molecular marker based on the 8-bp InDel observed in the promoter region was designed and named TaFlo2-Indel8 (Table 1). The forward and reverse primers of TaFlo2-Indel8 are located at −80 bp and 72 bp from the start codon, respectively. The PCR products of TaFlo2-Indel8 in the accessions with or without the 8-bp InDel have lengths of 153 bp and 145 bp, respectively. The bands of 153 bp and 145 bp were easily discriminated by polyacrylamide gel electrophoresis and represented the haplotypes *TaFlo2-A1a* and *TaFlo2-A1b*, respectively (Fig. 2c).

### Chromosomal location of *TaFlo2* genes

**Fig. 3:**
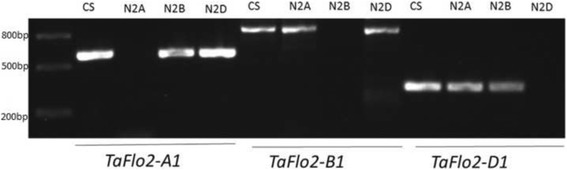
Assignment of *TaFlo2-A1*, *TaFlo2-B1* and *TaFlo2-D1* to wheat chromosomes 2A, 2B and 2D, respectively, by PCR mapping with the genomic DNA of Chinese Spring (CS) and derivative nulli-tetrasomic lines (N2AT2B, N2BT2A and N2DT2A). The size (kb) of DNA markers is shown to the left of the image

To assign chromosomal locations to *TaFlo2* genes, genome-specific primers and a set of Chinese Spring nulli-tetrasomic lines were used. The *TaFlo2* genes *TaFlo2-A1*, *TaFlo2-B1* and *TaFlo2-D1* were found to be located on chromosomes 2A, 2B and 2D (Fig. 3). The cloned sequences of *TaFlo2-A1*, *TaFlo2-B1* and *TaFlo2-D1* showed >99% similarity with 2AL, 2BL and 2DL scaffolds (IWGSC_chr2AL_ab_k71_contigs_longerthan_200_6436403, IWGSC_chr2BL_ab_k71_contigs_longerthan_200_7959819, IWGSC_chr2DL_ab_k71_contigs_longerthan_200_9909583). Further analysis revealed that the *TaFlo2-A1* gene was located on deletion bin ‘2AL1–0.85-1.00’.

### Association of *TaFlo2-A1* with thousand grain weight

To associate *TaFlo2-A1* with TGW, two natural populations, the Chinese Micro Core Collection (MCC) and the Pakistani collection, were used. In the MCC, the homozygous *TaFlo2-A1a* haplotype was found in 219 (83.5%) accessions, whereas the *TaFlo2-A1b* haplotype was found in 43 (16.5%) accessions. In the Pakistani wheat collection, the number of accessions carrying *TaFlo2-A1a* and *TaFlo2-A1b* were 85 (64.6%) and 45 (35.4%), respectively. Both in the MCC and Pakistani collections, the positive haplotype *TaFlo2A-A1b* had a lower frequency, which showed the scope of improving grain weight.

The difference in TGW between the haplotypes *TaFlo2-A1a* and *TaFlo2-A1b* was statistically significant in both populations (*P < 0.05*, Table 2). In the MCC, the mean difference in TGW between the groups of accessions having *TaFlo2-A1a* and *TaFlo2-A1b* was significant (*P* ≤ 0.05) across the 3 years of data. The mean differences in TGW between the two haplotypes in 2002, 2005 and 2006 were 7.00 ± 1.12 g, 7.80 ± 1.11 g and 8.40 ± 0.94 g, respectively. Consistent with the results of the MCC, the mean difference in TGW between groups of accessions having *TaFlo2-A1a* and *TaFlo2-A1b* was also significant (*P* ≤ 0.05) across both years of data in the Pakistani wheat collection. The values of the mean difference between the two haplotypes (*TaFlo2-A1a* and *TaFlo2-A1b*) in the Pakistani wheat population were 4.50 ± 0.71 g and 5.20 ± 0.72 g for 2009 and 2010, respectively. The phenotypic variance for TGW explained by *TaFlo2-A1* haplotypes was 6.19% in 2002, 7.76% in 2005 and 8.37% in 2006 in the MCC. In the Pakistani collection, the phenotypic variance for TGW explained by *TaFlo2-A1* haplotypes was 4.42% in 2009 and 5.11% in 2010 (Table 2). Moreover, to determine whether *TaFlo2-A1* also affects grain number per spike (GpS), an association analysis was performed for GpS in both populations. However, the differences in GpS between the haplotypes *TaFlo2-A1a* and *TaFlo2-A1b* were not significant in either population (*P* < 0.05, Table 2).

**Table 2:** Association of TGW and GpS with *TaFlo2-A1* in the Chinese Micro Core Collection and Pakistani wheat collections

Natural populations	Year (number of accessions)	*TaFlo2-A1a* Mean ± SE^a^ (number of accessions)		*TaFlo2-A1b* Mean ± SE (number of accessions)		Mean difference ± SE		PVE (%)^b^ TGW
		TGW	GpS	TGW	GpS	TGW	GpS	
Chinese Micro Core Collection	2002 (137)	33.6 ± 0.54(98)	50.8 ± 1.2(98)	40.6 ± 1.2(39)	49.7 ± 1.5(39)	7.0 ± 1.1**	1.06 ± 2.1^ns^	6.19
	2005 (169)	30.7 ± 0.52(128)	43.1 ± 0.8(128)	38.5 ± 1.1(41)	40.5 ± 1.1(41)	7.8 ± 1.1**	2.6 ± 1.5 ^ns^	7.76
	2006 (185)	32.8 ± 0.43(141)	51.4 ± 0.7(141)	41.2 ± 0.9(44)	48.6 ± 1.2(43)	8.4 ± 0.9**	2.7 ± 1.5 ^ns^	8.37
Pakistani collection	2009 (130)	40.6 ± 0.43(85)	46.6 ± 1.1(85)	45.1 ± 0.55(45)	44.4–1.8(45)	4.5 + 0.71**	2.2 ± 1.9 ^ns^	4.42
	2010 (130)	40.5 ± 0.47(85)	47.8 ± 1.2(85)	45.7 ± 0.46(45)	44.9 + 1.6(45)	5.2 ± 0.72**	2.9 ± 2.0 ^ns^	5.11

^**^indicates significant differences, and ^ns^ indicates non-significant differences (*P < 0.01*; Student’s t-test) among groups carrying different haplotypes

^a^Standard error

^b^Percentage of phenotypic variance explained by association analysis

Collectively, our data demonstrated that *TaFlo2-A1*, like the *OsFlo2* gene in rice, is associated with TGW in wheat. Furthermore, the lack of association of *TaFlo2-A1* with GpS suggests that the high TGW of the examined genotypes is primarily due to the positive haplotype (*TaFlo2-A1b*) for high TGW instead of loci for low number of kernels per spike and/or low grain yield.

### Expression of *TaFlo2-A1* is positively related to TGW

**Fig. 4:**
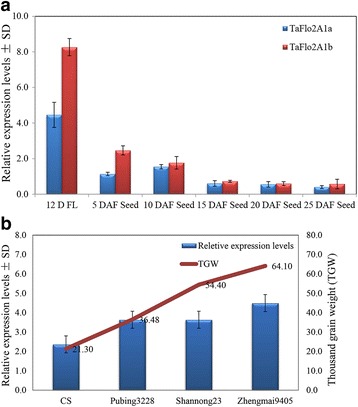
**a** Expression of *TaFlo2-A1* in flag leaves and developing grains. **b** Expression level of *TaFlo2-A1b* in cultivars with different TGW values at 5 DAF

To observe the contrasting effects of *TaFlo2-A1a* and *TaFlo2-A1b* on TGW at the gene expression level in flag leaves and developing grains, two polymorphic accessions were used. The expression level of *TaFLO2* was positively correlated with TGW, which is consistent with previous results in rice [27]. The haplotype *TaFlo2-A1a*, which exhibits low expression levels, represented the group of accessions that have low average TGW, and the haplotype *TaFlo2-A1b*, which exhibits high expression levels, represented the group of accessions that have high average TGW in both Chinese and Pakistani wheat populations. Quantitative RT-PCR assays showed that for both types of haplotypes, the expression level was maximum in 12-day-old flag leaves followed by expression in developing grains sampled at 5 DAF. However, the expression of both types of haplotypes decreased rapidly in the fertilized caryopses collected at 10, 15, 20 and 25 DAF. The expression level of *TaFlo2-A1b* was higher than that of *TaFlo2-A1a* at all tested stages but significantly differed only in flag leaves and developing grains at 5 DAF (Fig. 4a). Furthermore, the expression level was positively correlated in Chinese Spring and three cultivars (Pubing3228, Shannong23, and Zhengmai9405) in developing grains sampled at 5 DAF. The expression level was lowest in Chinese Spring (TGW, 21.3 g) and highest in the cultivar Zhengmai9405 (TGW, 64.1 g) (Fig. 4b). All these cultivars contained the positive haplotype *TaFlo2-A1b*. Together, these results suggested that the relative expression level of *TaFlo2-A1* was highest in flag leaves but started to decrease in developing grains. However, the expression in developing grains at 5 DAF was positively correlated with TGW in cultivars carrying the positive haplotype.

## Discussion

### Rice-wheat synteny-based gene cloning in wheat

The rice *OSFLO2* orthologs *TaFLO2-A1*, *TaFLO2-B1* and *TaFLO2-D1* were cloned, characterized and found to be located on homeologous chromosome group 2 in wheat. Sequence polymorphism observed in the promoter region of *TaFlo2-A1* was associated with TGW. Thus, *TaFLO2-A1* is a yield-related gene, and its manipulation could be useful for improving the grain yield potential of bread wheat. Many genes related to TGW and grain yield have been isolated and characterized in wheat using rice-wheat synteny [15]. The success of rice-wheat orthology-based gene cloning in wheat is due to high nucleotide and amino acid similarity between the corresponding orthologous genes. For example, with their respective rice orthologs, *TaTGW6* has 71% nucleotide and 68% amino acid similarity [16, 17]; *TaGW2* has 98% nucleotide and ~ 87% amino acid similarity [12]; *TaCKX6-D1* has 66% amino acid similarity [22]; *TaGS-D1* has 75.5% cDNA and 72.2% amino acid similarity [24]; and *TaGASR-A1* has 88% amino acid sequence similarity [26]. These data provide a genetic framework for marker-assisted selection (MAS) to pyramid positive alleles for TGW and yield during cultivar development. However, there are still many important genes that have been characterized in rice that are not being used as template for cloning their orthologs in wheat, e.g., *OsTB1* [36], *GW5* [37], *GS5* [38], *GW8* [39], *GW7/GL7* [40, 41], and *OsAGSW1* [42]. Thus, comparative genomics approaches between rice and wheat will remain useful in discovering orthologs of rice genes in wheat and will continue to enhance our understanding of the genetics of yield potential in wheat.

### The *TaFLO2-A1* gene is related to TGW in wheat

Map-based cloning using QTL mapping approaches is an important strategy to isolate loci and genes controlling genetic polymorphism [43]. However, progress on map-based cloning in wheat has been relatively slow compared to that in rice, and very few QTLs have been subjected to fine mapping in order to isolate candidate genes, mainly due to the complexity and large genome size of wheat.

In our study, *TaFlo2-A1* was found to be associated with TGW and explained from 4.42% (in the Pakistani collection) to 8.37% (in the MCC) of phenotypic variation. The TGW-related QTL identified on 2AL includes ‘*Xgwm339*-*Xbarc311*’ in 139 RILs between two hard red spring wheat lines [44]; *QTgw.ipk*-*2A* (*Xgwm372*) in 111 BC_2_F_3_ lines derived from the cross ‘Flair × XX86’ [45]; *QGwt.crc-2A* (*Xgwm558-Xgwm294*) in a double-haploid population generated from the cross ‘RL4452 × AC Domain’ [46]; *QTkw.sdau-2A* (*Xwmc181a*-*Xubc840c*) in 131 RILs derived from ‘Chuan 35050’ × ‘Shannong 483’ [13]; *QSZ.uaf-2A.1* (*Xwmc455*) in natural populations of 108 CIMMYT and Pakistani spring wheat accessions [47]; and *QTkw.hwwgr-2AL* (*Xgwm312*–IWA6090) in 127 RILs derived from ‘Ning7840’ × ‘Clark’ [42]. Furthermore, three QTL on 2AL that were stable across five trials were detected in the same MCC (262) used in present study [29].

These QTL on 2AL are located between *Xgwm71.2*/*Xgwm558* and *Xgwm294*, with an interval of 22 cM according to the consensus map of Somers [48] or 16.1 cM according to the ITMI map (http://wheat.pw.usda.gov/ggpages/SSRclub/GeneticPhysical/). From this TGW-QTL-rich region, only one gene, *TaCwi-A1*, has been isolated thus far between the *Xgwm 71.2* and *Xbarc15* deletion bin ‘C-2AL1–0.85’, which is adjacent to the centromere [18]. By integrating the information from the ITMI (http://wheat.pw.usda.gov/ggpages/SSRclub/GeneticPhysical/) and ‘Yu 8679 × Jing 411’ SSR + SNP [49] maps, the location of *TaFlo2-A1* was inferred on deletion bin ‘2AL1–0.85-1.00’. Hence, the *TaFlo2-A1* is a TGW-related gene located on the distal deletion bin of chromosome 2AL, and the molecular marker ‘TaFlo2-InDel8’ is an addition to the kit of wheat breeders for marker-assisted selection.

### Relationship between TGW and GpS

The relationship between the number of grains per spike (GpS) and the TGW was traditionally found as being negatively correlated [1–3]. However, the simultaneous selection of favored haplotypes for one plus neutral ones for the other or otherwise favored haplotypes for both traits has changed the correlations from negative to neutral or even positive [4]. Therefore, no significant correlation was observed between TGW and GpS in the collections of Chinese landraces [4], French winter wheat cultivars [5] or CIMMYT-derived spring cultivars and lines [6, 50], while significantly positive correlations were observed in Chinese modern cultivars [4].

In many genome-wide association studies (GWAS) for TGW and GpS, many loci were found to be associated with only one of the traits [4, 29, 50]. The favored haplotypes at these loci should increase the phenotypic value of the one trait without negatively affecting the phenotypic value of the others. Thus, selection of such QTL was likely a major factor in changing the relationship between TGW and GpS over time. Similarly, selection of the favored haplotype (*TaFlo2-A1b*) identified in this study would help to increase TGW without reducing the average GpS in wheat.

### Effect and putative mechanism of *TaFlo2-A1* in the determination of TGW

*TaFlo2-A1*, which is represented by two haplotypes in our study, was found to be significantly associated with TGW. Polymorphisms of an 8-bp InDel in the promoter and of five SNPs in the first intron were observed in *TaFlo2-A1*. The orthologs *TaFlo2-B1* and *TaFlo2-D1* lacked sequence variations associated with TGW (Fig. 2b). The association analysis of the Chinese Micro Core Collection (MCC) and Pakistani accessions indicated that *TaFlo2-A1b* was the superior haplotype for TGW. Nevertheless, some accessions that contained *TaFlo2-A1a* also had high TGW. This is mainly because the effect of *TaFlo2-A1* is likely masked by other genes associated with grain size [20].

In wheat, *TaFlo2-A1* consists of 23 exons that encode 1734 amino acids with four TPR motifs at the positions of 947 to 988, 1032 to 1072, 944 to 1017 and 1028 to 1106 amino acid residues. Furthermore, three mitochondrial CLU domains were also observed at 737–878, 50–162 and 357–401 amino acid residues (Fig. 1a). The rice *OsFlo2* gene also consists of 23 exons and encodes 1720 amino acids with three TPR motifs at the positions of 933–966, 975–1008, and 1017–1050 amino acid residues [27]. However, no mitochondrial CLU was reported in rice FLO2 by She et al. [27]. To confirm the absence of mitochondrial CLU in rice FLO2, we BLASTed the rice FLO2 protein (accession: CAE03171) in an NCBI CD search. The results of the rice FLO2 protein (accession: CAE03171) query using the NCBI CD system showed the presence of two mitochondrial CLU domains at the intervals of 52–124 and 721–863 and four TPRs at the intervals of 932–973, 1017–1057, 929–1002 and 1013–1091 amino acid residues (Fig. 1a). Thus, the prediction of wheat and rice FLO2 protein structure using NCBI CD indicates high similarity between their structures.

Flo2 is considered to be a member of a conserved gene family in plants [27]. *TaFlo2-A1* is abundantly expressed in flag leaves and in developing grains at 5 DAF stage, and the expression level of the positive haplotype (*TaFlo2-A1b*) was higher than that of the negative haplotype (*TaFlo2-A1a*). The phenomenon of higher expression of the positive *TaFlo2* haplotype is consistent with the results for rice *OsFlo2*, in which the overexpression of the positive haplotype significantly increases grain size [27]. In rice, the *flo2* mutation in the promoter and in the open reading frame hinders the expression of genes involved in the synthesis of starch and protein [27, 28]. In rice cultivars that have different genetic backgrounds, some *flo2* mutations negatively affect grain quality attributes such as amylose content, grain appearance and physiochemical properties despite maintaining or increasing grain size [27, 28]. Based on these similarities between *OsFlo2* and *TaFlo2-A1* at the sequence, structure and expression levels, the 8-bp InDel mutation in the *TaFlo2-A1* promoter likely regulates grain size by affecting the expression of genes involved in the synthesis of starch and protein in wheat grains. Therefore, the increased expression of *TaFlo2-A1* has a positive effect on grain yield but may have a negative effect on some grain quality attributes in wheat, which shall need further investigations.

The newly developed molecular marker ‘TaFlo2-InDel8’ is an addition to the kit of wheat breeder for marker-assisted selection. This study likely lead to additional investigations to unveil the exact regulatory mechanism of the *Flo2* gene in wheat.

## Conclusions

The *Flo2* orthologs in hexaploid wheat were cloned, and *TaFlo2-A1* was found to be associated with TGW but not with grain number per spike (GpS) in both the MCC and Pakistani wheat collections. The frequency of *TaFlo2-A1b* (positive haplotype) was low in commercial wheat cultivars; thus this haplotype can be selected to improve grain weight. This study likely lead to additional investigations to understand the regulatory mechanism of the *Flo2* gene in hexaploid wheat. The newly developed molecular marker ‘TaFlo2-InDel8’ could be incorporated into the kit of wheat breeders for use in marker-assisted selection.

## Additional file

Additional file 1: Figure S1. a. Part of *TaFlo2A1* sequence in eight accessions. The first four sequences are of accessions with low TGW and other four sequences are of accessions with high TGW. b. Part of *TaFlo2-B1* sequence in eight accessions. The first four sequences are of accessions with low TGW and other four sequences are of accessions with high TGW. c. Part of *TaFlo2-D1* sequence in eight accessions. The first four sequences are of accessions with low TGW and other four sequences are of accessions with high TGW. (PDF 377 kb)

## Abbreviations

Aa: Amino acid
ANOVA: Analysis of variance
Bp: Base pair
CIMMYT: the International Maize and Wheat Improvement Center
CS: Chinese Spring
CTAB: Cetyl trimethylammonium bromide
DAF: Days after flowering
Flo2: Floury endosperm2
GpS: Grain number per spike
GYS: Grain yield per spike
InDel: Insertion and deletion
ITMI: International Triticeae Mapping Initiative
MCC: Micro Core Collection
NCBI: National Center for Biotechnology Information
QTL: Quantitative trait loci
RIL: Recombinant inbred line
TGW: Thousand grain weight
TPR: Tetratricopeptide repeat
URGI: Unité de Recherche Génomique Info
