# Supplementary material for: TaFlo2-A1, an ortholog of rice Flo2, is associated with thousand grain weight in bread wheat (Triticum aestivum L.)
Source: BMC Plant Biol. 2017 Oct 16;17:164. doi: 10.1186/s12870-017-1114-3 (PMC5644068; doi:10.1186/s12870-017-1114-3)
Supplement: Additional file 1: Figure S1. — a. Part of TaFlo2A1 sequence in eight accessions. The first four sequences are of accessions with low TGW and other four sequences are of accessions with high TGW. b. Part of TaFlo2-B1 sequence in eight accessions. The first four sequences are of accessions with low TGW and other four sequences are of accessions with high TGW. c. Part of TaFlo2-D1 sequence in eight accessions. The first four sequences are of accessions with low TGW and other four sequences are of accessions with high TGW. (PDF 377 kb) [file 12870_2017_1114_MOESM1_ESM.pdf]

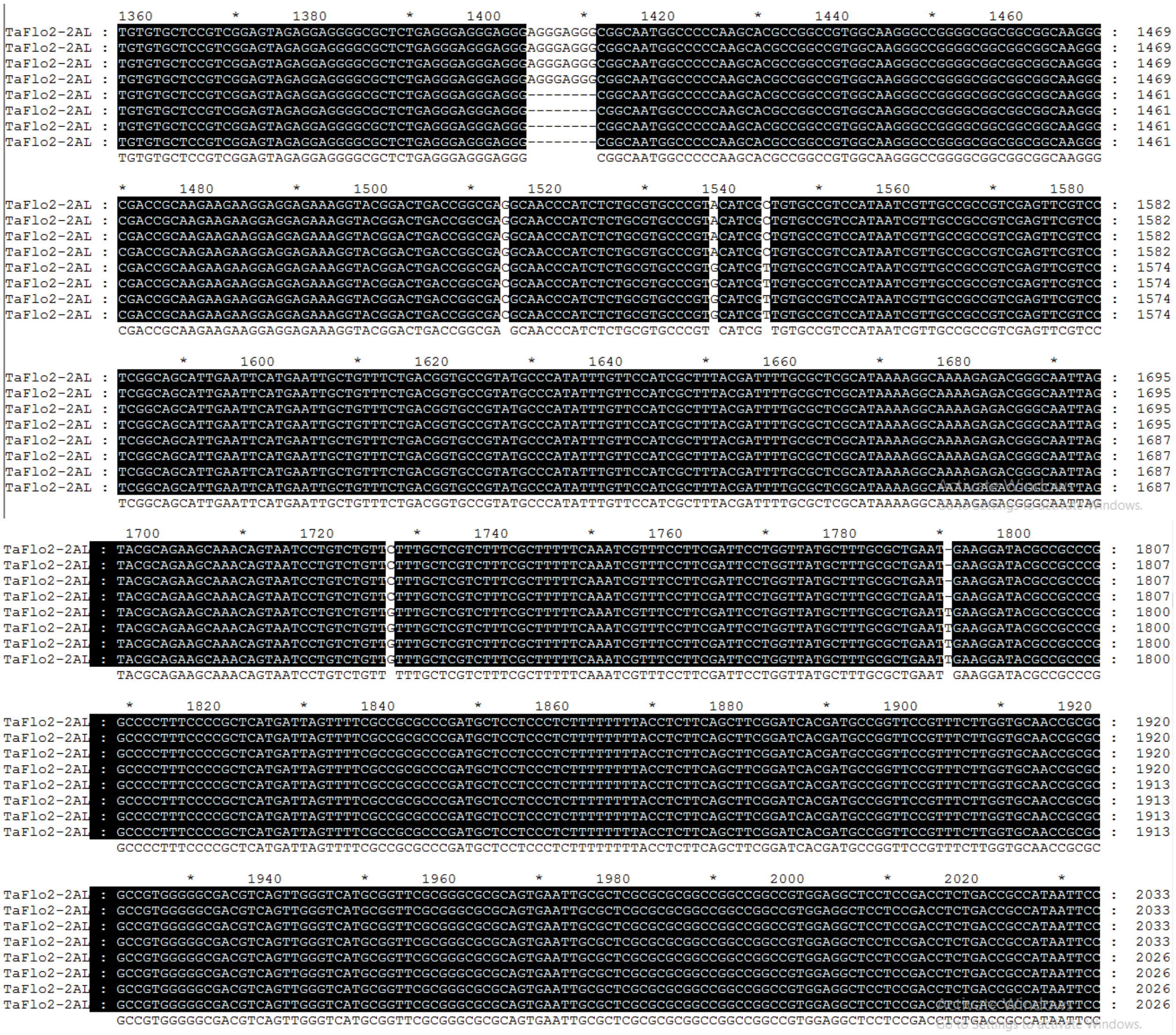

FigS1a. Part of *TaFlo2A1* sequence in eight accessions. The first four sequences are of accessions with low TGW and other four sequences are of accessions with high TGW.

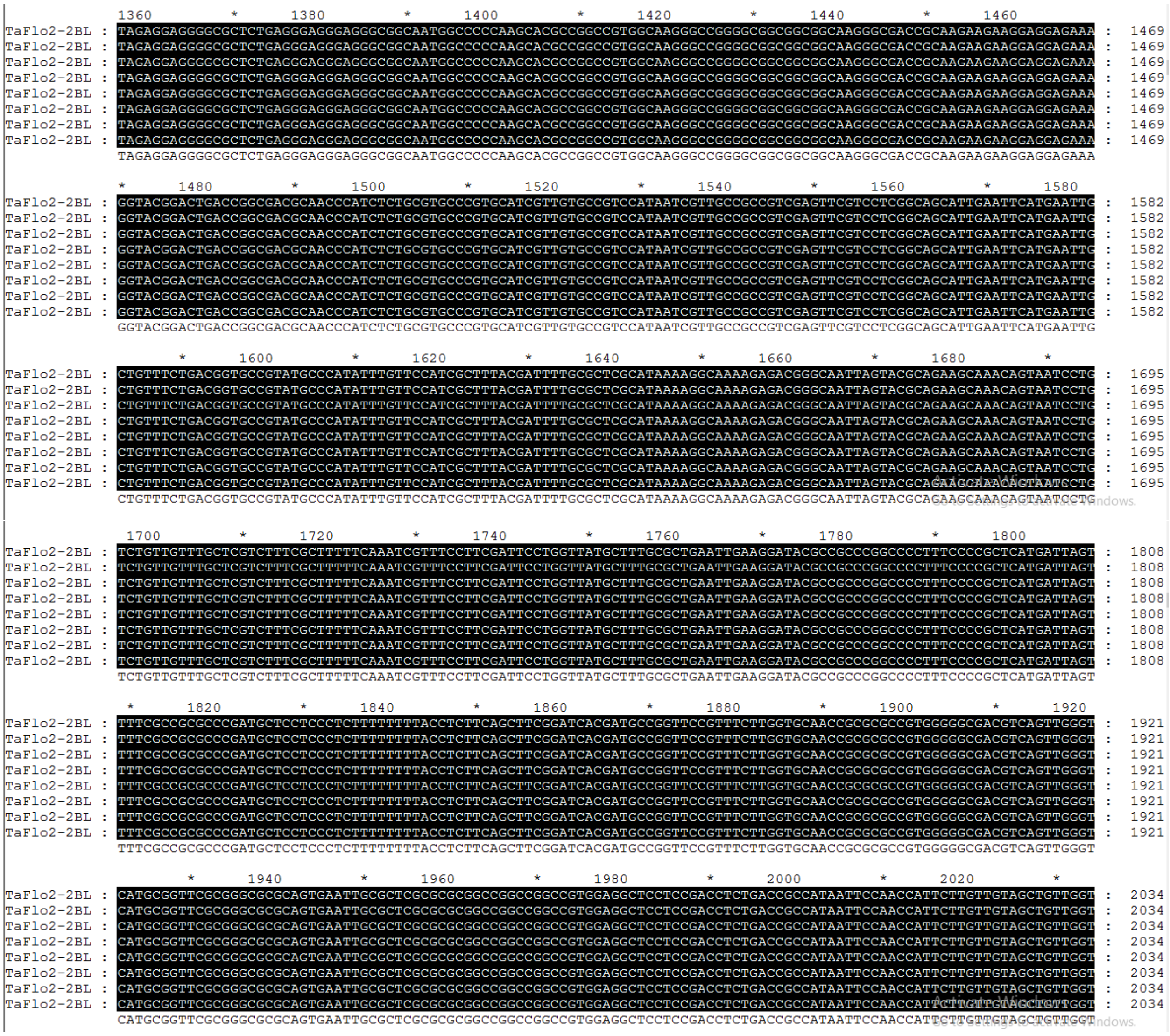

FigS1b. Part of *TaFlo2-B1* sequence in eight accessions. The first four sequences are of accessions with low TGW and other four sequences are of accessions with high TGW.

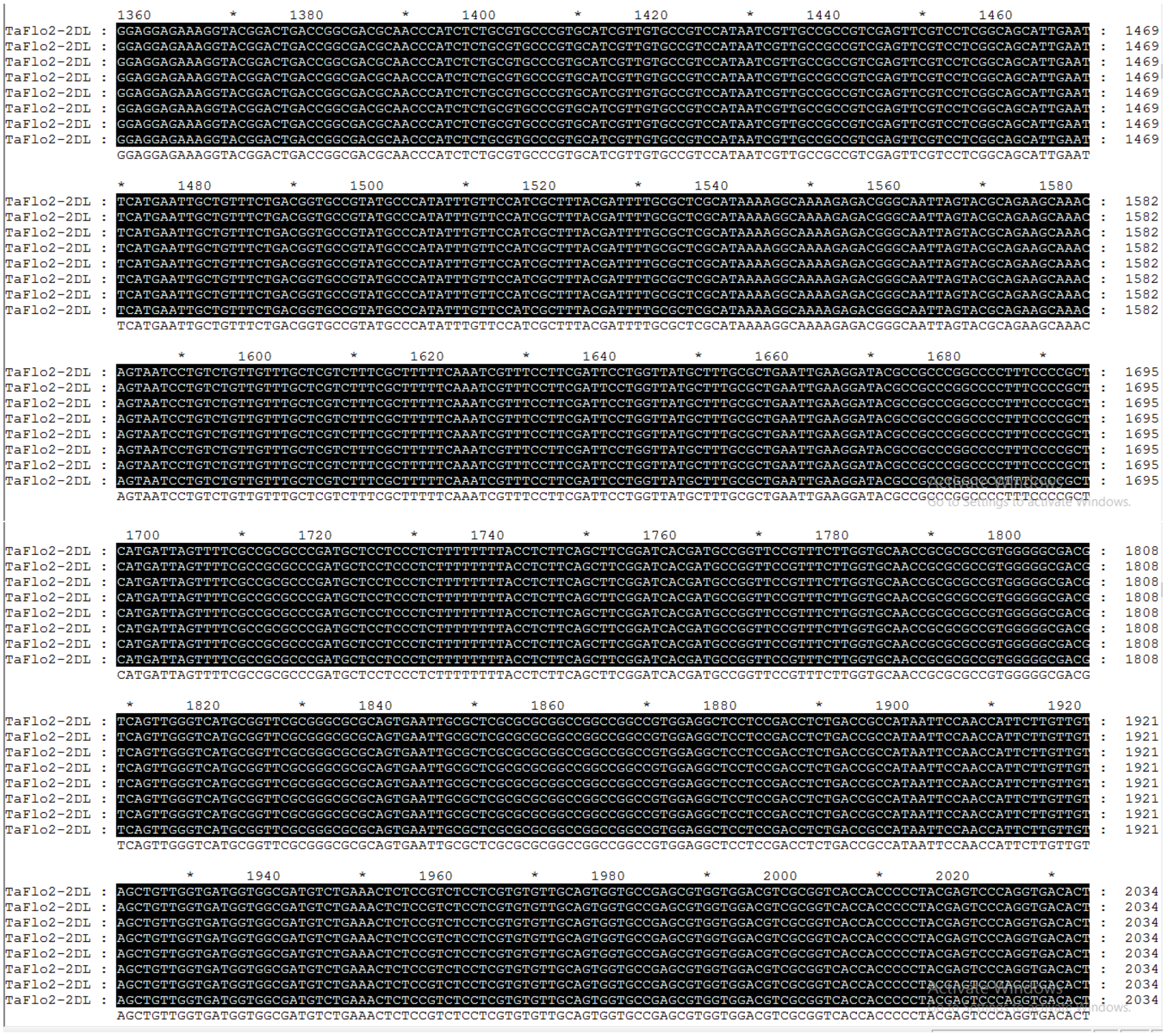

FigS1c. Part of *TaFlo2-D1* sequence in eight accessions. The first four sequences are of accessions with low TGW and other four sequences are of accessions with high TGW.
